# A Micromachined Piezoresistive Pressure Sensor with a Shield Layer

**DOI:** 10.3390/s16081286

**Published:** 2016-08-13

**Authors:** Gang Cao, Xiaoping Wang, Yong Xu, Sheng Liu

**Affiliations:** 1 School of Mechanical Science and Engineering, Huazhong University of Science and Technology, Wuhan 430074, China; caogang@hust.edu.cn (G.C.); xpwang@finemems.com (X.W.); 2 Department of Electrical and Computer Engineering, Wayne State University, Detroit, MI 48202, USA; yxu@eng.wayne.edu; 3 School of Power and Mechanical Engineering, Wuhan University, Wuhan 430072, China

**Keywords:** silicon pressure sensor, shield layer, stability

## Abstract

This paper presents a piezoresistive pressure sensor with a shield layer for improved stability. Compared with the conventional piezoresistive pressure sensors, the new one reported in this paper has an n-type shield layer that covers p-type piezoresistors. This shield layer aims to minimize the impact of electrical field and reduce the temperature sensitivity of piezoresistors. The proposed sensors have been successfully fabricated by bulk-micromachining techniques. A sensitivity of 0.022 mV/V/kPa and a maximum non-linearity of 0.085% FS are obtained in a pressure range of 1 MPa. After numerical simulation, the role of the shield layer has been experimentally investigated. It is demonstrated that the shield layer is able to reduce the drift caused by electrical field and ambient temperature variation.

## 1. Introduction

Micromachined silicon piezoresistive pressure sensors are receiving much attention due to their wide applications in the industrial control, automobile, aerospace, and biomedical fields [1,2,3]. Since the 1960s when the first piezoresistive pressure sensor was invented, many efforts have been made to improve the performance of silicon pressure sensors. Particularly, the stability of pressure sensors in harsh environments such as those encountered in the automotive, industrial, and military applications, is of great interest. French, Li and Guo et al. [4,5,6,7,8] developed pressure sensors based on a silicon-on-insulator (SOI) wafer. The oxide insulation layer prevents leakage between piezoresistors and the substrate, leading to a very low temperature coefficient of gauge factor (TCGF). Mehregany et al. [9] and Wu et al. [10] reported pressure sensors fabricated from silicon carbide, targeting applications in high-temperature and high-impact environments. Aryafar et al. [11] introduced a novel pressure sensor to reduce the thermal drift of sensitivity by implanting a polysilicon resistor in the pressure sensor. Peng et al. [12] fabricated a pressure sensor with an enhanced sensitivity by applying via etching technology. Lin et al. [13] and Godovitsyn et al. [14] proposed a surface micromachined pressure sensor by using polysilicon as a membrane and piezoresistors. Waber et al. [15] achieved a low hysteresis pressure sensor using a flip-chip bonded on copper springs. However, there are several disadvantages of the studies mentioned above, such as the high cost of SOI and silicon carbide wafers, low sensitivity of polysilicon piezoresistors, complex fabrication processes and so on. These limit the widespread use of these pressure sensors. Furthermore, intrinsic or extrinsic electrical fields can also cause sensor drift. This issue has not been adequately addressed. 

This paper reports a bulk micromachined silicon pressure sensor with a shield layer, aiming to reduce the electrical field and temperature impact on the sensor performance. Hoa et al. [16] investigated the influence of polysilicon as an electrical shield on the stability and reliability of piezoresistive pressure sensors. The electrical shield was made by polysilicon deposited on the insulation layer, and then connected to the supply voltage of the sensor. They concluded that the shield layer can stabilize the charge state around the piezoresistor, thereby improving the stability and reliability of the device. Although the benefit of the electrical shield is explicit, the additional polysilicon layer of the electrical shield may cause non-linearity and hysteresis problems. In order to avoid this disadvantage, the shield layer proposed in this paper is an n-type sheet in the silicon formed by phosphorus ion implantation above p-type piezoresistors. This additional layer improves the sensor stability by changing the dopant distribution and shielding the piezoresistors from electrical field interference.

## 2. Sensor Design and Fabrication

**Figure 1:**
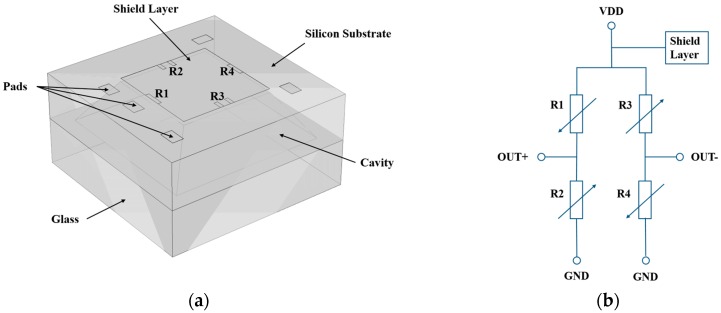
(**a**) Structure of the designed pressure sensor with a shield layer. (**b**) Equivalent circuit of the pressure sensor with a shield layer.

**Figure 2:**
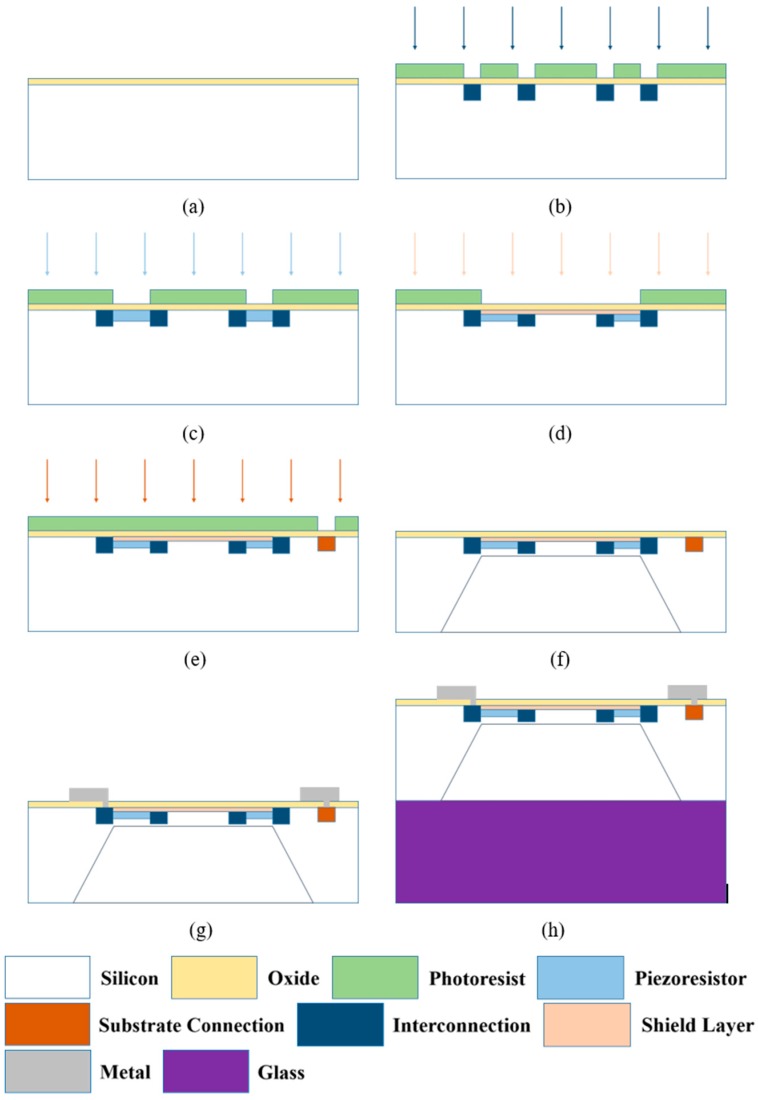
Process flow of the pressure sensor with a shield layer (**a**–**h**).

The pressure sensor with a shield layer and the corresponding equivalent electrical circuit are schematically shown in Figure 1a,b, respectively. As shown in Figure 1a, four p-type piezoresistors are placed at the centers of the diaphragm edges where the maximum stress occurs. The width and length of the piezoresistors are 15 μm and 75 μm, respectively. The lateral dimension of the sensing diaphragm is 1000 μm × 1000 μm and the thickness is 40 μm. In order to obtain a maximum sensitivity, all the longitudinal axes of the piezoresistors are aligned to the <110> direction. The n-type shield layer covers the piezoresistors and is directly connected to the highest potential of the sensor. The four piezoresistors are connected as a semi-closed Wheatstone-Bridge (Figure 1b). For comparison a conventional pressure sensor was fabricated, of which the piezoresistors are not covered by a shield layer. The simplified fabrication process of the pressure sensors is shown in Figure 2 and described below:

(a) Fabrication starts on a 500 μm double-side polished n-type (100) silicon wafer with a resistivity of 7.5 Ω·cm. First, 20 nm oxide is thermally grown at 1050 °C as ion implantation screen.
(b) P-type heavy boron implantation is carried out using photoresist mask to form the interconnection and lead-out structure at an energy of 80 keV with a dose of 1 × 10^16^ cm^−2^. After annealing, the doping concentration of this area is very high to maintain an ohmic contact with aluminum.
(c) Piezoresistors are formed by boron ion implantation at an energy of 130 keV. In order to make the piezoresistors of the two types of sensors have the same resistance value, the implantation dose of the sensor with a shield layer is 1.57 × 10^14^ cm^−2^, which is higher than 3 × 10^13^ cm^−2^, the dose for the sensor without a shield layer.
(d) N-type shield layer is formed by phosphorus ion implantation at an energy of 37 keV with a dose of 1.4 × 10^14^ cm^−2^. This process is not needed for the sensor without a shield layer.
(e) Substrate connection is formed by heavy phosphorus ion implantation at an energy of 60 keV with a dose of 8 × 10^15^ cm^−2^. After all the implantations are finished, a drive-in process is performed at 1050 °C.
(f) A cavity is formed by silicon anisotropic wet etching (75 °C, 45% KOH solution) at backside of the wafer. The diaphragm size and thickness are controlled by the cavity size and etching depth.
(g) After passivation, contact holes are opened at certain positions by reactive ion etching (RIE). Then a layer of 1 µm-thick aluminum is sputtered on the wafer and subsequently patterned to form a semi-closed Wheatstone-Bridge circuit.
(h) Pyrex^®^ 7740 glass (Insaco Inc., Quakertown, PA, USA) is bonded to the backside of the wafer under a vacuum environment. Finally, the wafer is diced into 2 mm × 2 mm pieces.

**Figure 3:**
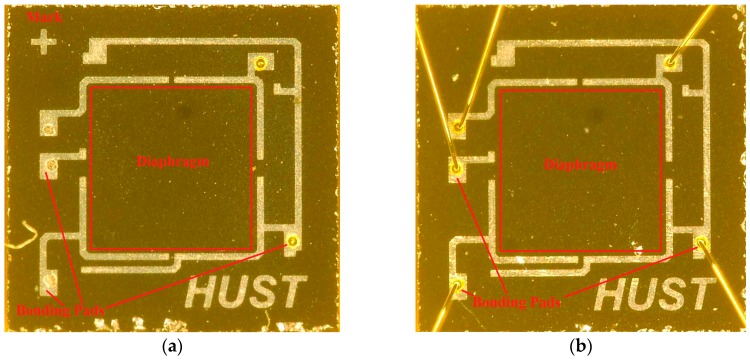
(**a**) Pressure sensor with a shield layer. (**b**) Pressure sensor without a shield layer.

Figure 3a,b shows micrographs of the two types of sensors fabricated. The red square marks the diaphragm. Note that piezoresistors and the shield layer are difficult to observe under the optical microscope. A cross mark (+) is placed on the upper-left corner of the chip to identify the sensor with a shield layer.

## 3. Electrical Field Stability Simulation

In many applications such as a tire pressure monitoring system (TPMS), pressure sensors may operate in harsh electromagnetic environments [17]. Both internal and external electrical fields can affect the operation of pressure sensors. The internal electrical field is generated by charges associated with the SiO_2_-Si system: fixed oxide charges, oxide trapped charges, mobile oxide charges and interface trapped charges. The charge concentration varies from 10^10^ to 10^12^ cm^−2^ depending on oxidation ambient, oxidation temperature, and cooling conditions [18]. For the piezoresistors, there is no difference between the external and internal electrical fields. Therefore, in order to simplify the models, only external electrical field is considered in this paper.

**Figure 4:**
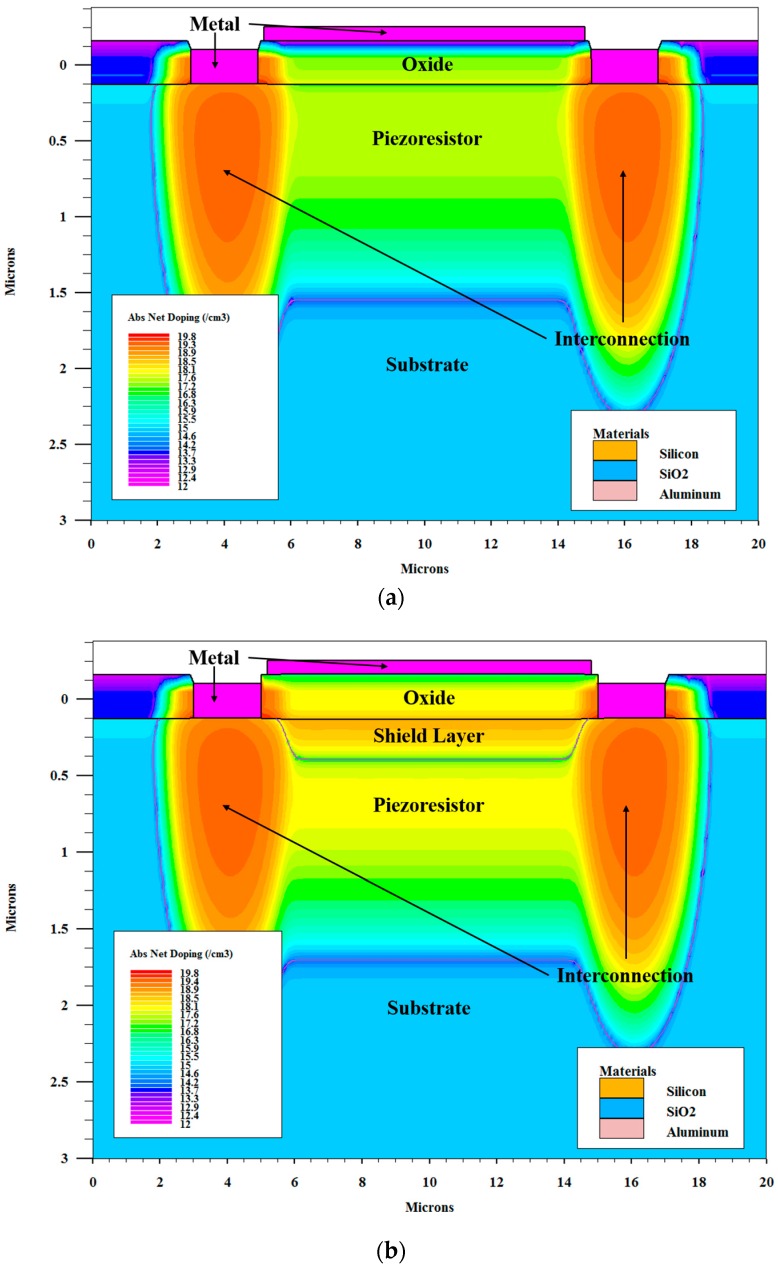
Simulated dopant distribution of pressure sensors (**a**) without a shield layer and (**b**) with a shield layer.

According to the process flow described above, process models are established for both types of pressure sensors using TCAD software to simulate the dopant distribution. To simplify these models, some processes such as wet etching, anodic bonding are not included. Figure 4a,b presents the result of the simulation. Figure 4a shows the cross section of the pressure sensor without a shield layer. The p-type piezoresistor is implanted in the n-type silicon substrate. The doping level of the p-type piezoresistor is much higher than that of the substrate. There is a p-n junction where the net doping level is very low between the piezoresistor and the substrate, which defines the junction depth of the piezoresistor. Figure 4b is the cross section of the pressure sensor with a shield layer. Compared with Figure 4a, the p-type piezoresistor is covered by the n-type shield layer. Therefore, there are two p-n junctions in the vertical direction, reducing the thickness of the piezoresistor. In order to emulate the external electrical field, a metal gate is added on the oxide above the piezoresistor in each model. When voltages are applied to the metal gates, electrical fields are induced in pressure sensors. In the electrical field stability simulations, 0 V, 5 V, 10 V, 15 V and 20 V voltages are applied to the metal gate in sequence. At each metal gate voltage, a DC sweep from 0 V to 5 V is carried out for the piezoresistor to obtain an I-V curve.

**Figure 5:**
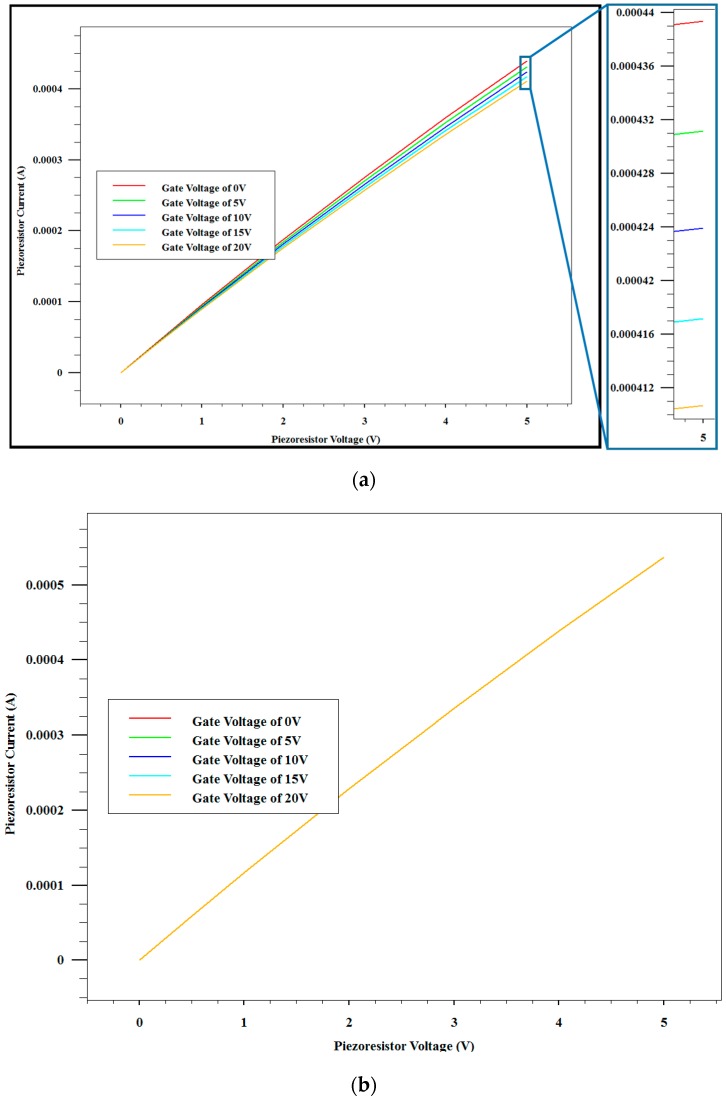
Simulated I-V characteristics of pressure sensors without (**a**) and with (**b**) a shield layer at different gate voltages.

Figure 5a,b presents the results of the I-V characteristics of the pressure sensors without and with a shield layer. Figure 5a clearly shows that the I-V curves of the piezoresistor are functions of the gate voltages if there is no shield layer. The piezoresistor current decreases from 0.44 mA to 0.41 mA when the gate voltage increases from 0 V to 20 V. In other words, the resistance of the piezoresistor increases by 7.4%, from 11.36 kΩ to 12.20 kΩ. The modulation by the electrical field can be clearly observed. On the contrary, the shield layer minimizes the impact of the electrical field on piezoresistors as shown in Figure 5b. Anderås observed this phenomenon in single crystalline silicon nanofilms [19]. It is reported that the interface of the oxide and silicon is in inversion when the metal gate is applied to an appropriate voltage. If the piezoresistor directly contacts with the interface, the inversion layer could reduce the thickness of the piezoresistor and accordingly increase the resistance. However, if the piezoresistor is covered by a shield layer, the inversion layer is separated. Therefore, the resistance will remain unchanged.

## 4. Temperature Stability Simulation

Sheet resistances of piezoresistors in these two models are approximately 1062 Ω/square and 986 Ω/square, respectively. Due to the existence of the shield layer, the thickness of the shielded piezoresistor is smaller than that of the unshielded piezoresistor. Thus, the dopants of the shielded piezoresistor are more concentrated than in the unshielded piezoresistor. It is also worth noting that the shielded piezoresistor has a higher doping level.

**Figure 6:**
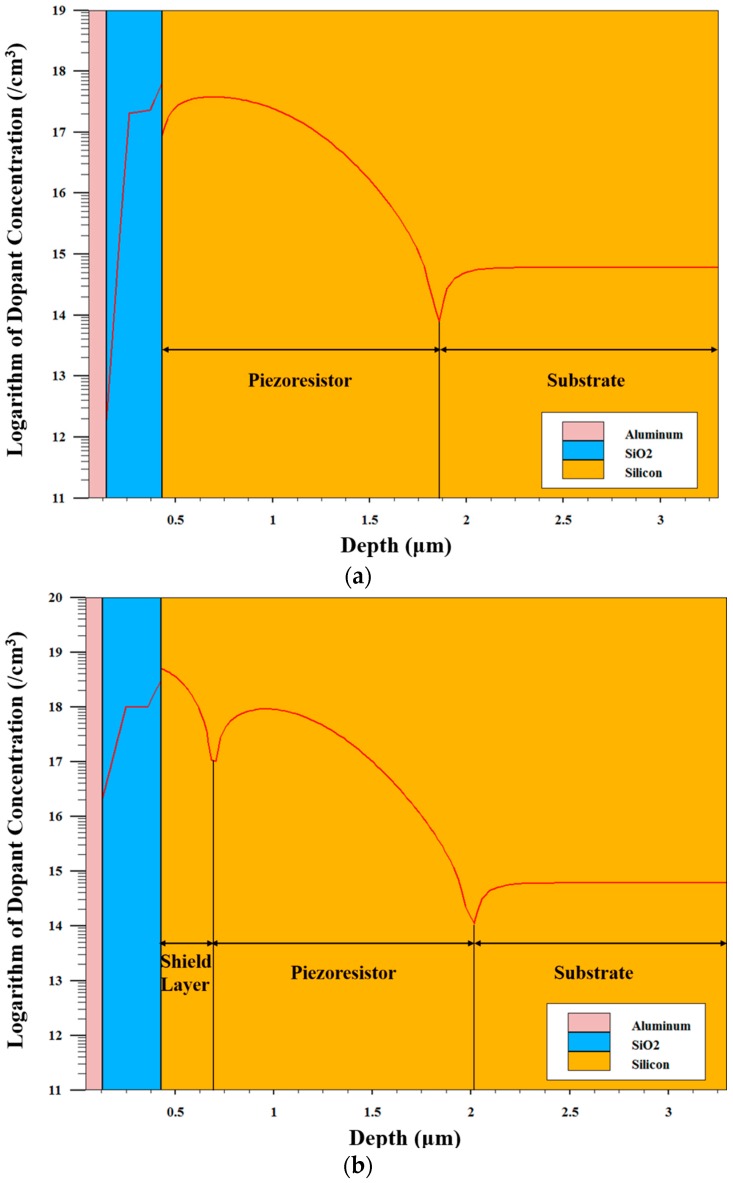
Dopant concentration versus depth without (**a**) and with (**b**) a shield layer.

The temperature coefficient of resistance (TCR) represents the temperature stability of the piezoresistor. Tufte and Stelzer discussed TCR in silicon [20]. The results presented in their work indicate that TCR of the piezoresistor is dependent on the doping concentration of the piezoresistor. Boukabache and Pons discussed the doping effects on the thermal behavior of silicon resistor as well [21]. They showed that a heavy doping could reduce the TCR of the silicon resistor. Figure 6a,b presents the plots of net doping concentration along the depth of pressure sensors without and with a shield layer respectively, which are extracted from Figure 4a,b. As shown in Figure 6a, the piezoresistor uncovered by a shield layer extends from the boundary of the substrate and oxide to 1.43 µm deep, and the highest level of the doping concentration is about 3 × 10^17^ cm^−3^. As a comparison, the vertical range of the piezoresistor covered by a shield layer is from 0.32 µm deep to 1.58 µm deep, as shown in Figure 6b. The highest level of the doping concentration is about 1 × 10^18^ cm^−3^. These differences make the piezoresistors exhibit different temperature sensitivities.

Sridhar and Foster have given a method to calculate TCR based on doping concentrations [22]. The piezoresistor is treated as a number of thin piezoresistor layers stacking together. In every thin piezoresistor layer, the doping concentration is uniform. The sheet resistance of piezoresistor is defined as:

(1) $$R(T)=\frac{1}{G(T)}$$

where *G*(*T*) is sheet conductance, and is given by:

(2) $$G(T)=\sum_{i}q\cdot \mu_{p}(T,N_{tot_{i}})\cdot p(T,N_{net_{i}})\cdot \left[D_{i}-D_{i-1}\right]$$

where *q* is electronic charge, $\mu_{p}\left(T,N_{tot_{i}}\right)$ is the hole mobility as a function of temperature *T* and total active impurity concentration $N_{tot_{i}}$ at the depth *D_i_*, *D_i_* − *D_i_*_–1_, is the thickness of the piezoresistor layer, and $p\left(T,N_{net_{i}}\right)$ represents the hole concentration at temperature *T* and net active concentration $N_{net_{i}}$ at the depth *D_i_*. $N_{tot_{i}}$ and $N_{net_{i}}$ of shielded and unshielded piezoresistors can be obtained from Figure 6a,b, respectively. $\mu_{p}\left(T,N_{tot_{i}}\right)$ and $\left(T,N_{net_{i}}\right)$ are given by Equations (3) and (4):

(3) $$\mu_{p}(T,N_{tot_{i}})=54.3\cdot T_{n}{\left(T\right)}^{-0.57}+\left[\frac{1.36\cdot 10^{8}\cdot T_{n}{\left(T\right)}^{-2.23}}{1+\left[\frac{N_{tot_{i}}}{2.35\cdot 10^{17}\cdot T_{n}{\left(T\right)}^{2.4}}\right]\cdot 0.88\cdot T_{n}{\left(T\right)}^{-0.146}}\right]$$

(4) $$p(T,N_{net})=N_{net}+\frac{{N_{i}}^{2}}{N_{net}}$$

where *T_n_*(*T*) = *T*/300, *N_i_* is intrinsic carrier concentration, which is defined as:

(5) $$N_{i}=3.39\cdot 10^{15}\cdot T^{3/2}\cdot \exp(-\frac{E_{g}(0)}{2k_{0}T})\cdot \exp(\frac{4.9\cdot 10^{-4}\cdot T}{2k_{0}\cdot (T+655)})$$

where *E_g_*(0) is the band gap of silicon at *T* = 0 K, which is 1.169 eV; *k*_0_ is Boltzmann’s constant. *R*(*T*) can be fitted to a second order polynomial in temperature *T*:

(6) $$R(T)\approx R_{0}\cdot \left[1+\alpha \cdot (T-273)+\beta \cdot {\left(T-273\right)}^{2}\right]$$

where *R*_0_ is the sheet resistance at room temperature (273 K), *α* and *β* are the first and second order TCR respectively. *R*_0_, *α* and *β* are numerically computed using MATLAB, and the results are shown in Table 1.

As the results in Table 1 illustrate, the first and second order TCRs of the pressure sensor with the shield layer is smaller than those without the shield layer. This indicates that the shield layer is effective in enhancing the pressure sensor’s temperature stability.

## 5. Packaging and Testing

**Figure 7:**
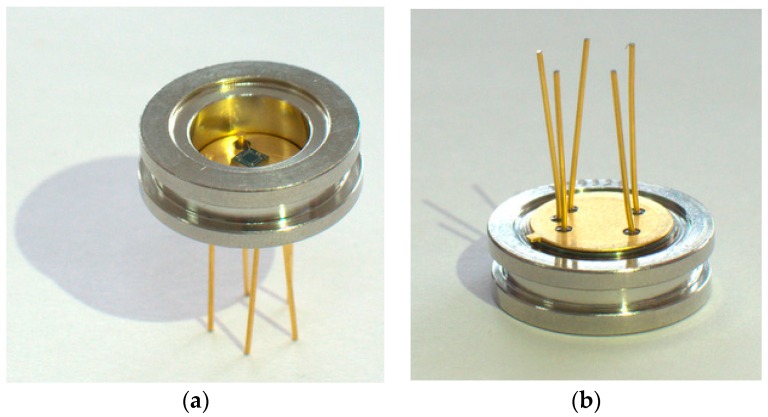
(**a**) Top view and (**b**) bottom view of a packaged pressure sensor.

**Figure 8:**
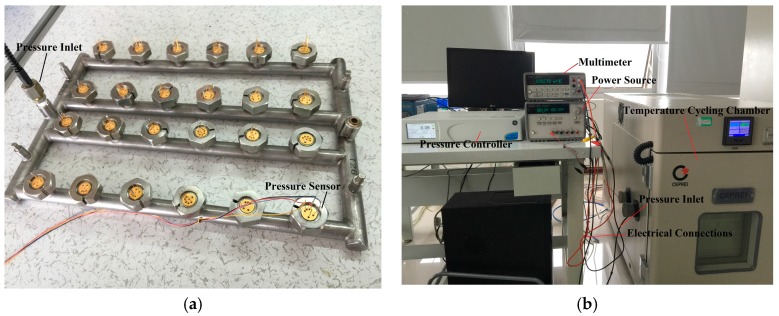
(**a**) A custom-made fixture and (**b**) testing apparatus for pressure sensor characterization.

For experimental characterization, the pressure sensors are packaged as shown in Figure 7. The five pads of each pressure sensor are wire bonded to five legs of the package respectively. After being coated with soft silicone gel, the pressure sensors are coupled to a fixture as shown in Figure 8a which can then be placed in a controlled environment. The testing apparatus is shown in Figure 8b. The pressure is regulated by a pressure controller (GE Druck PACE5000, Burlington, VT, USA) and the temperature is controlled by a temperature cycling chamber (CEEC-WSJ-60C, CEPREI, Guangzhou, China). The output of the pressure sensors is measured by an Agilent E34401A multimeter (Santa Clara, CA, USA). An Agilent E3631A is used to provide 5 V DC voltage to power the Wheatstone bridge. 

The impact of electrical field on the piezoresistor was tested first. The resistance of the piezoresistors is measured as the gate voltage increases from 0 V to 20 V. The testing results are summarized in Table 2. It clearly shows that the resistance of the unshielded piezoresistor strongly depends on the gate voltage. The resistance increases about 12.4% when the gate voltage increases from 0 V to 20 V, slightly larger than the simulation results. It may be caused by the difference of the insulation layer between the simulation model and the actual one. The insulation between the metal gate and the silicon is set as silicon oxide in the simulation. However, the real sensors use silicon nitride, whose dielectric constant is larger than that of silicon oxide. Therefore, the density of the charges on the metal gate is higher than the simulation one. The higher density of the charges can introduce a higher strength electrical field. The resistance increases more when the electrical field strength gets higher. On the contrary, the resistance of the shielded piezoresistor remains constant when the gate voltage changes. 

**Figure 9:**
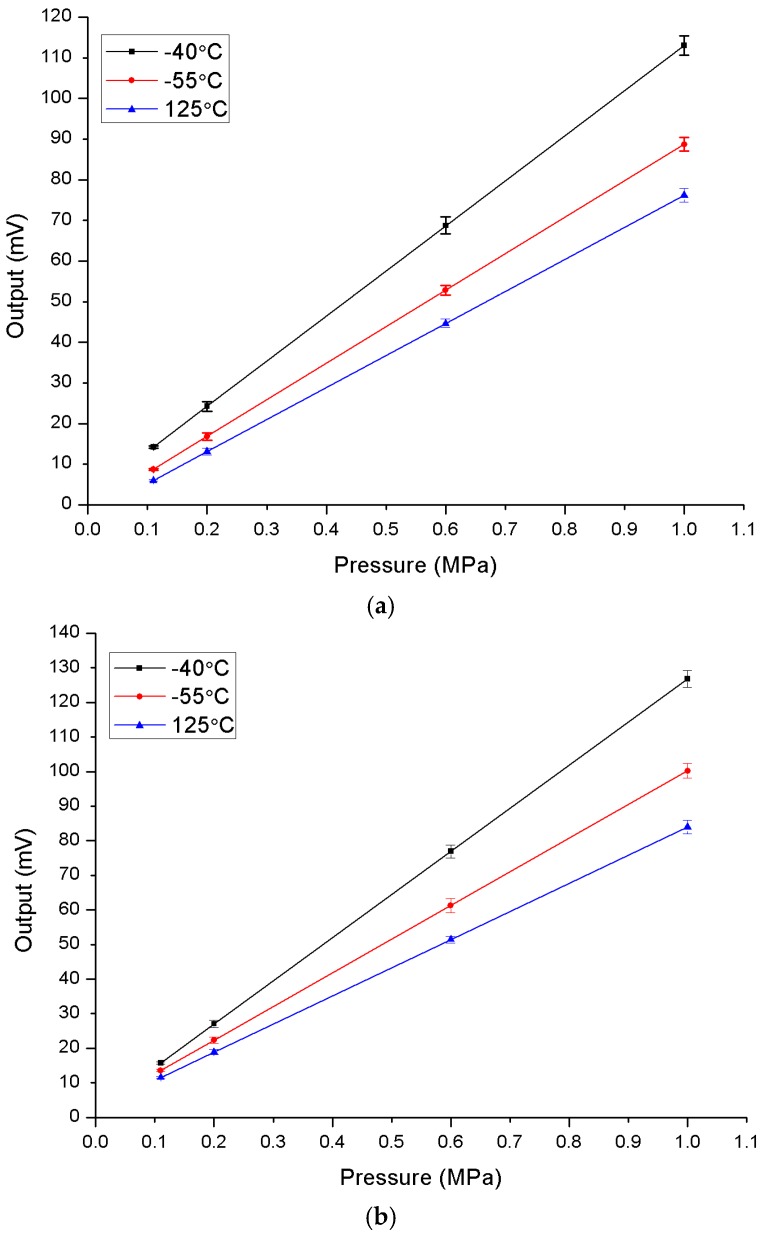
Characteristics of the pressure sensor with (**a**) and without (**b**) a shield layer at different temperatures.

Next, the pressure sensitivity of fabricated pressure sensors is characterized at −40 °C, 55 °C, and 125 °C. The output data, including the calculated sensitivity and non-linearity of the pressure sensors, are listed in Table 3 and Table 4. Figure 9a,b shows the output curves of the pressure sensors at different temperatures. The testing results show that both types of pressure sensor have a high sensitivity and a low non-linearity. As shown in Table 3 and Table 4, the sensitivity of the pressure sensor without a shield layer is 0.016 mV/V/kPa at 125 °C and increases to 0.025 mV/V/kPa at −40 °C. The sensitivity drift is 56%. As a comparison, the sensitivities of the pressure sensor with a shield layer are 0.016 mV/V/kPa at 125 °C and 0.022 mV/V/kPa at −40 °C, respectively. The sensitivity drift is 37%. A lower sensitivity drift reveals that the shield layer plays an important role in the temperature stability.

**Figure 10:**
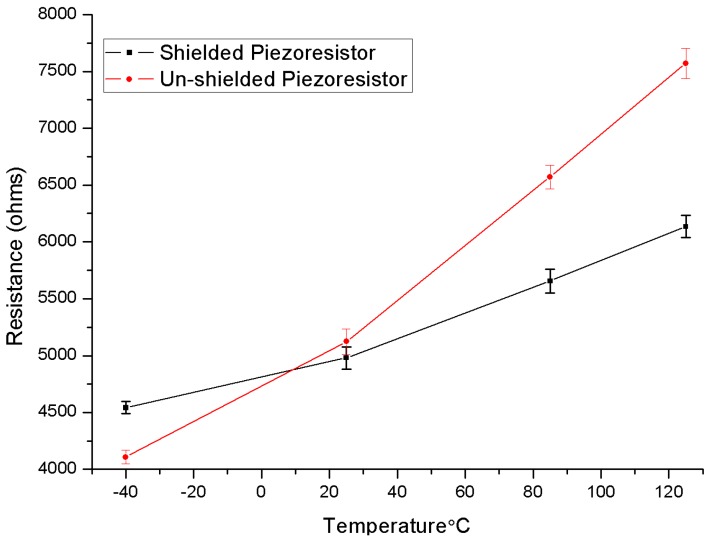
Temperature sensitivity of shielded and un-shielded piezoresistors.

The TCRs of pressure sensors with and without a shield layer are investigated at the same time. Table 5 shows the measured resistance and extracted TCRs of two types of piezoresistors. It can be observed that the shield layer effectively reduces the first order and second order TCRs of the piezoresistor. Figure 10 shows the measured data in the form of a line graph. 

The resistance of the shielded piezoresistor increases more slowly than the un-shielded one as the temperature increases, indicating a smaller temperature sensitivity. The temperature stability difference between experiment and simulation can be caused by a number of factors. First, the actual doping profiles cannot precisely match the simulation ones shown in Figure 6. The actual doping profile is not only affected by the implant energy and dose, but also depends heavily on the thickness of the implantation screen oxide and the annealing process. Second, the leakage of the p-n junctions between the piezoresistors and the substrate may affect the temperature characteristics of the piezoresistor.

## 6. Conclusions

A bulk-micromachined pressure sensor with improved stability has been successfully developed. The improvement is achieved by an n-type shield layer fabricated above p-type piezoresistors. The n-type layer can shield the electrical field and change the dopant distribution of the pressure sensor. Therefore, piezoresistors covered by the shield layer are less sensitive to the electrical field and temperature than uncovered ones. Both simulation and experimental results show that the resistance of piezoresistors not covered by the shield layer is a function of the metal gate voltage, however, the shield layer can make the piezoresistor insensitive to the metal gate voltage. Furthermore, both simulation and experimental results reveal that the first order and second order TCRs of shielded piezoresistors are smaller than the ones of un-shielded piezoresistors. The pressure sensor with a shield layer exhibits a sensitivity of 0.022 mV/V/kPa and a maximum non-linearity of 0.085%FS. The sensitivity drift of the pressure sensor with a shield layer is 37% in a temperature range from −40 °C to 125 °C, which is lower than the one without a shield layer.

## Figures and Tables

**Table 1:** TCR computation results by using MATLAB.

Pressure Sensor	Sheet Resistance *R*_0_	TCR	
	(Ω/square)	*α* (ppm/°C)	*β* (ppm/°C)
With Shield Layer	970	1342	0.15
Without Shield Layer	1057	1612	3.18

**Table 2:** Resistance of the piezoresistors vs. gate voltage.

Resistance (Ω)	Gate Voltage (V)				
	0	5	10	15	20
Unshielded Piezoresistor	5464	5833	6015	6081	6147
Shielded Piezoresistor	5043	5042	5042	5043	5042

**Table 3:** Outputs of pressure sensor with a shield layer versus temperature (*T*) and pressure (*P*).

	*P*	0.11 MPa	0.2 MPa	0.6 MPa	1 MPa	Sensitivity (mV/V/kPa)	Non-Linearity (%FS)
*T*							
−40 °C		14.20	24.23	68.71	112.98	0.022	0.085%
55 °C		8.68	16.80	52.81	88.70	0.018	0.023%
125 °C		6.00	13.12	44.67	76.14	0.016	0.013%

**Table 4:** Outputs of pressure sensor without a shield layer versus temperature (*T*) and pressure (*P*).

	*P*	0.11 MPa	0.2 MPa	0.6 MPa	1 MPa	Sensitivity (mV/V/kPa)	Non-Linearity (%FS)
*T*							
−40 °C		15.81	27.04	76.92	126.80	0.025	0.043%
55 °C		13.59	22.35	61.28	100.22	0.019	0.054%
125 °C		11.61	18.93	51.47	84.01	0.016	0.005%

**Table 5:** Test data and calculated TCRs.

Pressure Sensor	Measured Resistance (Ω)				Measured TCRs	
	−40 °C	25 °C	85 °C	125 °C	*α* (ppm/°C)	*β* (ppm/°C)
Shielded Piezoresistor	4542	4980	5655	6135	1576	5.4
Unshielded Piezoresistor	4109	5124	6572	7572	3656	10.0
